# Predictive power of extubation failure diagnosed by cough strength: a systematic review and meta-analysis

**DOI:** 10.1186/s13054-021-03781-5

**Published:** 2021-10-12

**Authors:** Jun Duan, Xiaofang Zhang, Jianping Song

**Affiliations:** 1 Department of Respiratory and Critical Care Medicine, The First Affiliated Hospital of Chongqing Medical University, Youyi Road 1, Yuzhong District, Chongqing, 400016 China; 2 Department of Geriatric Respiratory, People’s Hospital of Wenjiang District, Kangtai Road 86, Wenjiang District, Chengdu, Sichuan Province 611130 China; 3 Department of Rehabilitation Medicine, The First Affiliated Hospital of Chongqing Medical and Pharmaceutical College, Nancheng Road 301, Nan’an District, Chongqing, 400060 China

**Keywords:** Ventilator weaning, Weak cough, Sensitivity, Specificity

## Abstract

**Background:**

The predictive power of extubation failure diagnosed by cough strength varies by study. Here we summarise the diagnostic power of extubation failure tested by cough strength.

**Methods:**

A comprehensive online search was performed to select potentially eligible studies that evaluated the predictive power of extubation failure tested by cough strength. A manual search was also performed to identify additional studies. Data were extracted to calculate the pooled sensitivity, specificity, positive likelihood ratio (LR), negative LR, diagnostic odds ratio (DOR), and area under the receiver operating characteristic curve (AUC) to evaluate the predictive power of extubation failure.

**Results:**

A total of 34 studies involving 45 study arms were enrolled, and 7329 patients involving 8684 tests were analysed. In all, 23 study arms involving 3018 tests measured cough peak flow before extubation. The pooled extubation failure was 36.2% and 6.3% in patients with weak and strong cough assessed by cough peak flow, respectively. The pooled sensitivity, specificity, positive LR, negative LR, DOR, and AUC were 0.76 (95% confidence interval [CI]: 0.72–0.80), 0.75 (0.69–0.81), 2.89 (2.36–3.54), 0.37 (0.30–0.45), 8.91 (5.96–13.32), and 0.79 (0.75–0.82), respectively. Moreover, 22 study arms involving 5666 tests measured the semiquantitative cough strength score (SCSS) before extubation. The pooled extubation failure was 37.1% and 11.3%, respectively, in patients with weak and strong cough assessed by the SCSS. The pooled sensitivity, specificity, positive LR, negative LR, DOR, and AUC were 0.53 (95% CI: 0.41–0.64), 0.83 (0.74–0.89), 2.50 (1.93–3.25), 0.65 (0.56–0.76), 4.61 (3.03–7.01), and 0.74 (0.70–0.78), respectively.

**Conclusions:**

Weak cough is associated with increased extubation failure. Cough peak flow is superior to the SCSS for predicting extubation failure. However, both show moderate power for predicting extubation failure.

**Supplementary Information:**

The online version contains supplementary material available at 10.1186/s13054-021-03781-5.

## Background

The use of a spontaneous breathing trial (SBT) has been recommended to help determine whether a patient can be weaned from mechanical ventilation (MV) [1–3]. After a successful SBT, extubation is recommended. However, 10–20% of patients who successfully complete an SBT experience extubation failure [4]. Compared to patients experience successful extubation, those who experience extubation failure are more likely to die in hospital [5, 6]. Evidence shows that early identification of patients at high risk for extubation failure and early application of preventive strategies (e.g. noninvasive ventilation or the use of a high-flow nasal cannula) can reduce hospital mortality [7, 8]. Therefore, the key question is how to identify patients at high risk for extubation failure.

Weak cough is a predictor of extubation failure. It can be measured by cough peak flow [9–17]. In some studies, patients with successful extubation had a higher cough peak flow than those who experienced extubation failure [9–16]. However, another study reported that cough peak flow did not differ between patients who experienced extubation success and failure [17]. In addition, cough strength can also be measured by the semiquantitative cough strength score (SCSS) [18–21]. Given the inconsistent results found by different studies and the use of multiple methods to measure cough strength, we reviewed the literature systematically and performed a meta-analysis to assess the efficacy of diagnostic tests that use cough strength for the early detection of extubation failure.

## Methods

### PICO statement

P-patient: adult patients were under MV through endotracheal intubation. I-index test: cough strength was measured in all included patients. C-complement: an SBT was given to all included patients who were deemed ready to be liberated from MV. O-outcome: the efficacy of cough strength for predicting extubation failure was estimated.

### Search techniques and selection criteria

This systematic review and meta-analysis was performed in conformance with the Preferred Reporting Items for Systematic Reviews and Meta-analysis statement [22]. We searched pertinent research published before June 2021 in PubMed, Web of Science, the Cochrane library, and some Chinese databases (CBM, Wanfang Data, and CNKI) without any language limitations. We also did manual searches of the reference lists of included articles to identify additional relevant articles. The studies were searched with the following key words: (“weak cough” OR “ineffective cough” OR “cough peak flow” OR “cough peak expiratory flow” OR “cough strength”) and (“ventilator weaning” OR “wean from mechanical ventilation” OR “weaning from mechanical ventilation” OR “liberation from mechanical ventilation” OR “liberate from mechanical ventilation” OR “withdrawal of mechanical ventilation” OR “extubation failure” OR “postextubation failure” OR “postextubation respiratory failure” OR “reintubation”).

Studies were enrolled based on the following inclusion criteria: (1) only adult patients with an endotracheal tube were involved, (2) an SBT was completed before extubation, (3) cough strength was assessed before extubation, and (4) data were available for calculating outcomes (true positive [TP], false positive [FP], false negative [FN], and true negative [TN]). The following works were excluded: (1) reviews, case reports, editorials, letters, and conference abstracts; (2) articles with no available data for patients with weak cough; and (3) articles without a definition of extubation failure. Extubation failure included reintubation, death, or the use of noninvasive ventilation due to postextubation respiratory failure.

### Data extraction and evaluation of quality

All studies were independently selected by two investigators (JD and XFZ). Any discrepancies were resolved by consensus. If the researchers failed to reach a consensus, a third investigator (JPS) reviewed the data in question. The first author’s name; publication year; study region; sample size; methods of assessing cough strength; cut-off value; definition of weak cough; and number of patients with TP, FP, FN, and TN were collected. If numbers of TP, FP, FN, and TN were unavailable, we communicated with the corresponding author to obtain these data. The Quality Assessment of Diagnostic Accuracy Studies 2 was used to assess the quality of the enrolled articles [23].

### Statistical analysis

The data were analysed with RevMan 5.3, Meta-Disc 1.4, and Stata SE 15.0. The pooled diagnostic odds ratio (DOR), sensitivity, specificity, positive likelihood ratio (LR), negative LR, and area under the receiver operating characteristic curve (AUC) were calculated by TP, FP, FN, TN. Sensitivity = true positives/(true positives + false negatives). Specificity = true negatives/(true negatives + false positives). True positives were patients with ineffective cough who failed extubation. False negatives were patients with effective cough who failed extubation. True negatives were patients with effective cough who were successfully extubated. False positives were patients with ineffective cough who were successfully extubated. Diagnostic power was good, moderate, and poor if the AUC was more than 0.8, between 0.7 and 0.8, and less than 0.7, respectively [24]. Deeks’ funnel plot was used to detect publication bias. If publication bias was present, a sensitivity analysis was performed to explore why.

Spearman’s correlation coefficient is used to detect threshold effects. *I*^2^ is used to describe heterogeneity. *I*^2^ ≥ 50% represents significant heterogeneity. A fixed effects model was used if no heterogeneity was observed. A random effects model was selected if significant heterogeneity was observed. Possible sources of heterogeneity were explored through a meta-regression analysis.

## Results

### Characteristics of the included studies

**Fig. 1:**
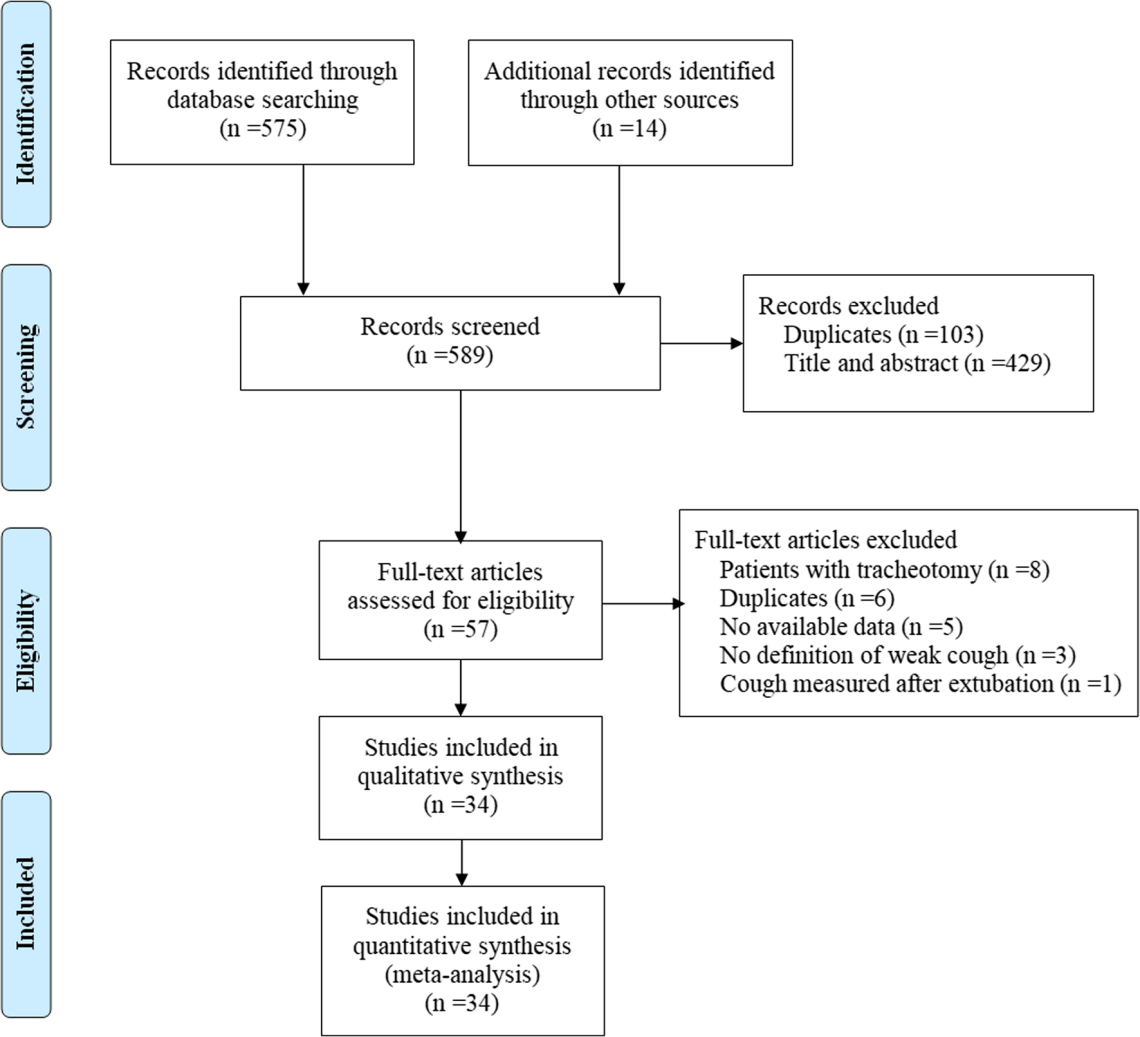
Flowchart of study selection

A total of 575 studies were obtained using the search strategy, and 14 studies were identified from other sources (Fig. 1). After screening titles and abstracts and reviewing full papers, we enrolled 34 studies involving 45 study arms in this meta-analysis [9–21, 25–45]. A total of 7329 patients involving 8684 tests were analysed. The characteristics of the study arms are summarised in Table 1. A total of 23 study arms involving 3018 tests measured cough peak flow before extubation. The pooled extubation failure was 36.2% and 6.3%, respectively, among patients with weak and strong cough assessed by cough peak flow (Additional file 1: Figure 1). Spearman’s correlation coefficient was 0.034 (*p* = 0.88), indicating no threshold effect. Four subgroups of studies measured cough peak flow. Details are reported in Table 2 and Additional file 12: Text 1.

**Table 1:** Characteristics of the included studies

Author	Year	Country	Design	Method of SBT	Measurement of cough strength	Definition of weak cough	Total tests	TP	FP	FN	TN	Time and definition of extubation failure	
Beuret	2009	France	Prospective	T-piece	Voluntary CPF tested with an external flowmeter	CPF ≤ 35 L/min	130	11	34	3	82	48 h	Reintubation
Duan	2014a	China	Prospective	PSV	Voluntary CPF tested with an external flowmeter	CPF ≤ 62.4 L/min	115	17	34	3	61	72 h	Reintubation
Duan	2014b	China	Prospective	PSV	#Involuntary CPF tested with an external flowmeter	CPF ≤ 49.8 L/min	115	14	32	6	63	72 h	Reintubation
Gao	2009a	China	Prospective	PSV/CPAP	Voluntary CPF tested with a ventilator	CPF ≤ 58.5 L/min	200	20	55	8	117	72 h	Reintubation/death
Gao	2009b	China	Prospective	PSV/CPAP	SCSS (strong, moderate, weak)	Weak	200	13	3	15	169	72 h	Reintubation/death
Salam	2004a	USA	Prospective	T-piece/PSV	Voluntary CPF tested with an external flowmeter	CPF ≤ 60 L/min	88	11	25	3	49	72 h	Reintubation
Salam	2004b	USA	Prospective	T-piece/PSV	WCT	Negative	88	10	36	4	38	72 h	Reintubation
Smailes	2013	UK	Prospective	T-piece	Voluntary CPF tested with an external flowmeter	CPF ≤ 60 L/min	125	10	7	7	101	48 h	Reintubation
Smina	2003	USA	Prospective	T-piece/PSV	Voluntary CPF tested with an external flowmeter	CPF ≤ 60 L/min	111	9	25	4	73	72 h	Reintubation
Su	2010a	China	Prospective	T-piece/PSV/CPAP	#Involuntary CPF tested with an external flowmeter	CPF ≤ 58.5 L/min	150	25	25	7	93	Hospital stay	Reintubation
Su	2010b	China	Prospective	T-piece/PSV/CPAP	SCSS (strong, weak, no cough)	Weak or no cough	150	25	47	7	71	Hospital stay	Reintubation
Khamiees	2001a	China	Prospective	T-piece/PSV/CPAP	SCSS (grade 0 to 5)	Grade 0 to 2	100	10	14	8	68	72 h	Reintubation
Khamiees	2001b	China	Prospective	T-piece/PSV/CPAP	WCT	Negative	100	9	16	9	66	72 h	Reintubation
Huang	2013	China	Retrospective	T-piece/PSV	SCSS (effective and ineffective)	Ineffective	119	23	27	4	65	7 d	Reintubation
Gobert	2017	France	Prospective	PSV	Voluntary CPF tested with a ventilator	CPF ≤ 60 L/min	92	7	24	4	57	48 h	Reintubation/death
Liu	2014	China	Prospective	PSV	Voluntary CPF tested with a ventilator	CPF ≤ 60 L/min	102	8	36	7	51	48 h	Reintubation
Duan	2015a	China	Prospective	PSV	SCSS (grade 0 to 5)	Grade 0 to 2	186	16	52	12	106	72 h	Reintubation
Duan	2015b	China	Prospective	PSV	Voluntary CPF tested with an external flowmeter	CPF ≤ 60 L/min	186	23	71	5	87	72 h	Reintubation
Bai	2017a	China	Prospective	PSV	Voluntary CPF tested with an external flowmeter	CPF ≤ 56.4 L/min	126	11	14	4	97	72 h	Reintubation
Bai	2017b	China	Prospective	PSV	Voluntary CPF tested with an external ventilator	CPF ≤ 56 L/min	126	11	16	4	95	72 h	Reintubation
Xiao	2018	China	Prospective	PSV	Voluntary CPF tested with an external flowmeter	CPF ≤ 60 L/min	139	15	36	7	81	72 h	Reintubation
Duan	2017	China	Prospective	PSV	Voluntary CPF tested with an external flowmeter	CPF ≤ 70 L/min	356	61	119	15	161	7 d	Reintubation
Thille	2015	France	Prospective	PSV	SCSS (grade 0 to 4)	Grade 0 to 2	223	10	15	20	178	7 d	Reintubation
Kutchak	2015	Brazil	Prospective	T-piece	$Involuntary CPF tested with an external flowmeter	CPF ≤ 80 L/min	135	35	20	10	70	48 h	Reintubation
Almeida	2020a	Brazil	Prospective	T-piece/PSV	Voluntary CPF tested with an external flowmeter	CPF ≤ 45 L/min	81	23	3	10	45	48 h	Reintubation
Almeida	2020b	Brazil	Prospective	T-piece/PSV	#Involuntary CPF tested with an external flowmeter	CPF ≤ 60 L/min	81	29	4	4	44	48 h	Reintubation
Almeida	2020c	Brazil	Prospective	T-piece/PSV	$Involuntary CPF tested with an external flowmeter	CPF ≤ 55 L/min	81	30	7	3	41	48 h	Reintubation
Aziz	2018	Egypt	Prospective	Not reported	SCSS (grade 0 to 5)	Grade 0 to 2	80	18	3	19	40	72 h	Reintubation
Vivier	2019a	France	Prospective	T-piece	SCSS (ineffective, moderate, and effective)	Ineffective	181	6	7	27	141	7 d	Reintubation/death
Vivier	2019b	France	Prospective	T-piece	Voluntary CPF tested by an external flowmeter	CPF ≤ 60L/min	160	18	74	10	58	7 d	Reintubation/death
Wang	2019	China	Retrospective	Not reported	SCSS (with or without spontaneous cough)	Without spontaneous cough	86	19	0	6	61	Hospital stay	Reintubation
Ma	2018	China	Retrospective	Not reported	SCSS (strong, weak, no cough)	Weak or no cough	108	11	6	8	83	48 h	Reintubation
Frutos-Vivar	2006	Canada	Prospective	T-piece/PSV/CPAP	SCSS (poor, moderate, or excellent)	Poor	900	33	178	88	601	72 h	Reintubation
Jaber	2018	France	Prospective	T-piece/PSV/CPAP	SCSS (weak and strong)	Weak	1505	116	811	32	546	48 h	Reintubation
Dos	2017	Brazil	Prospective	T-piece/PSV	SCSS (grade 0 to 5)	Grade 0 to 2	311	8	21	35	247	48 h	Reintubation
Michetti	2018	USA	Prospective	PSV/CPAP	SCSS (not strong and strong)	Not strong	464	11	142	24	287	96 h	Reintubation
Abbas	2018	Egypt	Prospective	Not reported	SCSS (grade 0 to 5)	Grade 0 to 2	90	7	11	19	53	48 h	Reintubation/NIV
Norisue	2020	Japan	Prospective	PSV	Voluntary CPF tested with a ventilator	CPF ≤ 50 L/min	252	8	62	4	178	72 h	Reintubation
Sanson	2018	Italy	Prospective	Not reported	SCSS (strong, weak, no cough)	Weak or no cough	205	21	121	5	58	ICU stay	Reintubation/NIV
Wang	2009a	China	Prospective	PSV	SCSS (grade 0 to 5)	Grade 0 to 2	68	9	9	11	39	72 h	Reintubation
Wang	2009b	China	Prospective	PSV	WCT	Negative	68	10	11	10	37	72 h	Reintubation
Elkholy	2021	Egypt	Prospective	PSV	WCT	Negative	150	21	24	1	104	72 h	Reintubation
Lu	2010	China	Prospective	PSV	Voluntary CPF tested with an external flowmeter	CPF ≤ 29.35 L/min	19	7	4	0	8	72 h	Reintubation
Liang	2019	China	Prospective	PSV	#Involuntary CPF tested with a ventilator	CPF ≤ 71.15 L/min	48	8	4	2	34	48 h	Reintubation/death
Thille	2020	France	Prospective	T-piece/PSV	SCSS (grade 0 to 4)	Grade 0 to 1	284	11	16	45	212	7 d	Reintubation/death

Letters a, b, and c after the year (e.g. 2014a and 2014b) represent different arms of a given study

^#^Cough was stimulated with 2 mL normal saline

^$^Cough was stimulated with suction catheter

CPF = cough peak flow, SCSS = semiquantitative cough strength score, WCT = white card test, TP = true positive, FP = false positive, FN = false negative, TN = true negative, NIV = noninvasive ventilation, SBT = spontaneous breathing trial, PSV = pressure support ventilation, CPAP = continuous positive airway pressure

Sensitivity = true positives/(true positives + false negatives). Specificity = true negatives/(true negatives + false positives)

True positives were patients with ineffective cough who failed extubation. False negatives were patients with effective cough who failed extubation

True negatives were patients with effective cough who were successfully extubated. False positives were patients with ineffective cough who were successfully extubated

**Table 2:** Summary of the outcomes of different subgroups

	Measurement of cough peak flow				Measurement of semiquantitative cough strength score		
	Voluntary CPF	Involuntary CPF	CPF measured with an external flowmeter	CPF measured with a ventilator	SCSS (grade 0 to 4/5)	WCT	Other#
No. of study arms	17	6	18	5	8	4	10
Total cases	2282	529	2023	718	1342	406	3918
Total tests	2408	610	2300	718	1342	406	3918
Pooled sensitivity	0.73 (0.68–0.78)	0.82 (0.73–0.88)	0.77 (0.72–0.81)	0.72 (0.60–0.81)	0.36 (0.26–0.48)	0.70 (0.44–0.88)	0.59 (0.41–0.47)
Pooled specificity	0.72 (0.65–0.79)	0.82 (0.74–0.88)	0.74 (0.67–0.81)	0.77 (0.69–0.84)	0.87 (0.80–0.91)	0.74 (0.61–0.84)	0.83 (0.62–0.64)
Pooled positive LR	2.7 (2.1–3.4)	4.5 (2.9–7.0)	3.0 (2.3–4.0)	3.1 (2.1–4.6)	2.7 (2.1–3.6)	2.7 (1.5–4.8)	3.5 (1.5–8.2)
Pooled negative LR	0.37 (0.31–0.45)	0.22 (0.14–0.35)	0.31 (0.25–0.39)	0.37 (0.25–0.55)	0.73 (0.64–0.84)	0.40 (0.18–0.90)	0.49 (0.33–0.73)
Pooled DOR	7 (5–10)	21 (9–48)	10 (6–15)	9 (4–18)	4 (3–5)	7 (2–25)	7 (2–21)
Pooled AUC	0.76 (0.72–0.79)	0.89 (0.86–0.91)	0.80 (0.77–0.84)	0.77 (0.73–0.81)	0.70 (0.65–0.73)	0.78 (0.74–0.82)	0.75 (0.71–0.79)

CPF = cough peak flow, SCSS = semiquantitative cough strength score, WCT = white card test, LR = likelihood ratio, DOR = diagnostic odds ratio, AUC = area under the receiver operating characteristic curve

^#^Includes strong, moderate, and weak; strong, weak, and no cough; effective and ineffective; with or without spontaneous cough; excellent, moderate, and poor; strong and weak; strong and not strong; and effective, moderate, and ineffective

Assessment of the SCSS before extubation was performed in 22 study arms involving 5666 tests. The pooled extubation failure was 37.1% and 11.3%, respectively, among patients with weak and strong cough assessed by the SCSS (Additional file 2: Figure 2). Spearman’s correlation coefficient was 0.450 (*p* = 0.04), indicating the presence of a threshold effect. Three subgroups of studies measured the SCSS. Details are reported in Table 2 and Additional file 12: Text 1.

### Quality assessment and publication bias

**Fig. 2:**
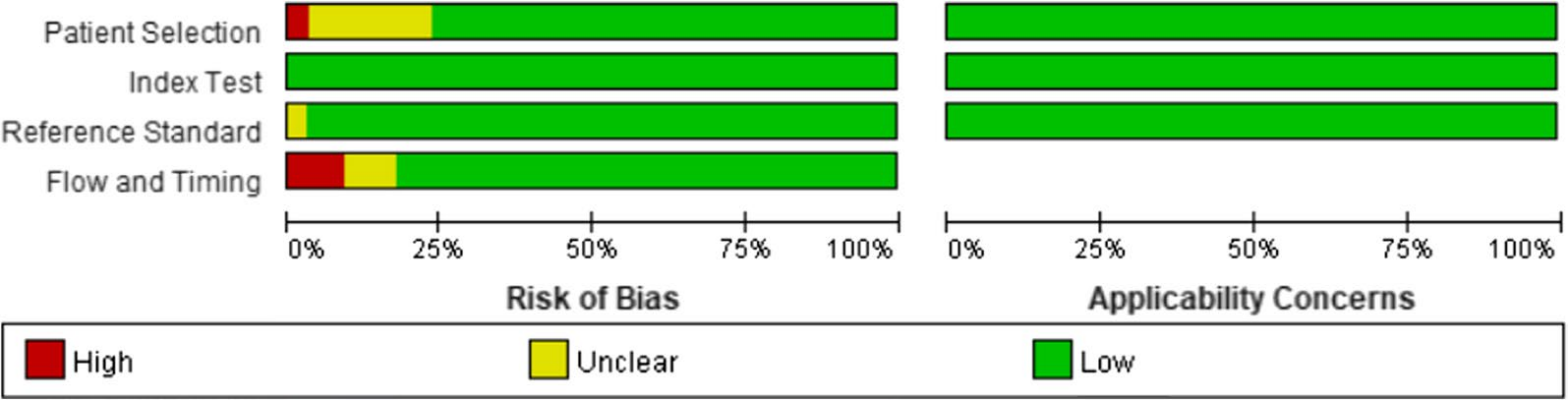
Quality Assessment of Diagnostic Accuracy Studies criteria for the included studies

The quality of the included studies is summarised in Fig. 2. The main high risk of bias was the time between the removal of the endotracheal tube and extubation failure. The majority of studies judged extubation failure at a prespecified time after extubation, detailed in Table 1, except for four studies. Three study arms collected data on extubation failure during hospitalisation after extubation. And one study arm collected data on extubation failure during the ICU stay after extubation. Additional file 3: Figure 3 shows the lack of publication bias among studies that used cough peak flow to predict extubation failure (*p* = 0.41). Additional file 4: Figure 4 shows the presence of publication bias among studies that used the SCSS to predict extubation failure (*p* = 0.02). The sensitivity analysis showed that excluding Frutos–Vivar et al.’s study [34] negated the publication bias (*p* = 0.07). The sensitivity analysis also showed that the pooled DOR ranged from 4.08 to 5.02 and the pooled AUC ranged from 0.71 to 0.75 when one study was omitted (Additional file 5: Figure 5).

### Accuracy of extubation failure diagnosed by cough peak flow

**Fig. 3:**
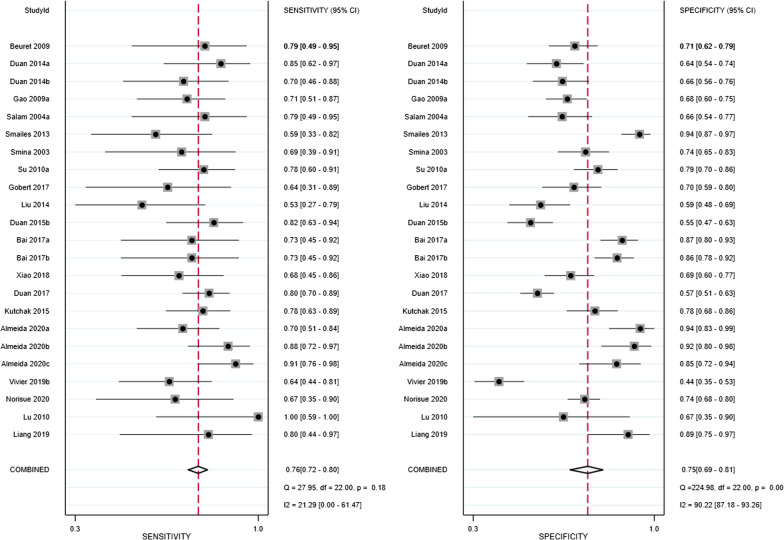
Forest plot of sensitivity and specificity in the diagnosis of extubation failure tested by cough peak flow. CI = confidence interval

**Fig. 4:**
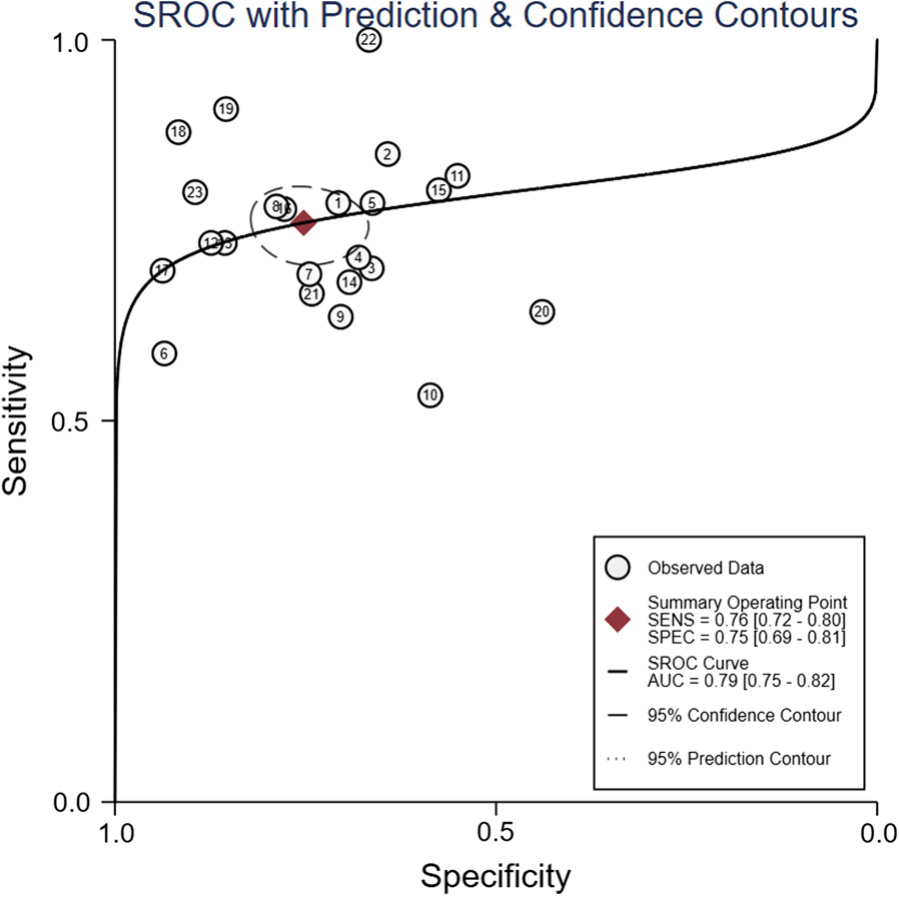
Summary receiver operator characteristic (SROC) curve in the prediction of extubation failure tested by cough peak flow. SENS = sensitivity, SPEC = specificity, AUC = area under the receiver operating characteristic curve. Numbers 1 to 23 represent the study arms (Beuret 2009, Duan 2014a, Duan 2014b, Gao 2009a, Salam 2004a, Smailes 2013, Smina 2003, Su 2010a, Gobert 2017, Liu 2014, Duan 2015b, Bai 2017a, Bai 2017b, Xiao 2018, Duan 2017, Kutchak 2015, Almeida 2020a, Almeida 2020b, Almeida 2020c, Vivier 2019b, Norisue 2020, Lu 2010, and Liang 2019)

The pooled sensitivity and specificity were 0.76 (95% confidence interval [CI]: 0.72–0.80) and 0.75 (0.69–0.81), respectively (Fig. 3). Meta-regression analyses indicated that sensitivity and specificity did not vary by publication year, country, assessment of voluntary or involuntary cough peak flow, assessment of cough peak flow with an external flowmeter or a ventilator, different cut-off values, number of cases in the study arm, time to extubation failure after the removal of the endotracheal tube, or definition of extubation failure (Additional file 6: Figure 6). The pooled positive LR and negative LR were 2.89 (95% CI: 2.36–3.54) and 0.37 (0.30–0.45), respectively (Additional file 7: Figure 7). The pooled DOR was 8.91 (95% CI: 5.96–13.32; Additional file 8: Figure 8). The AUC was 0.79 (95% CI: 0.75–0.82) when cough peak flow was used to predict extubation failure (Fig. 4). The results of subgroup analyses are summarised in Table 2.

### Accuracy of extubation failure diagnosed by the SCSS

**Fig. 5:**
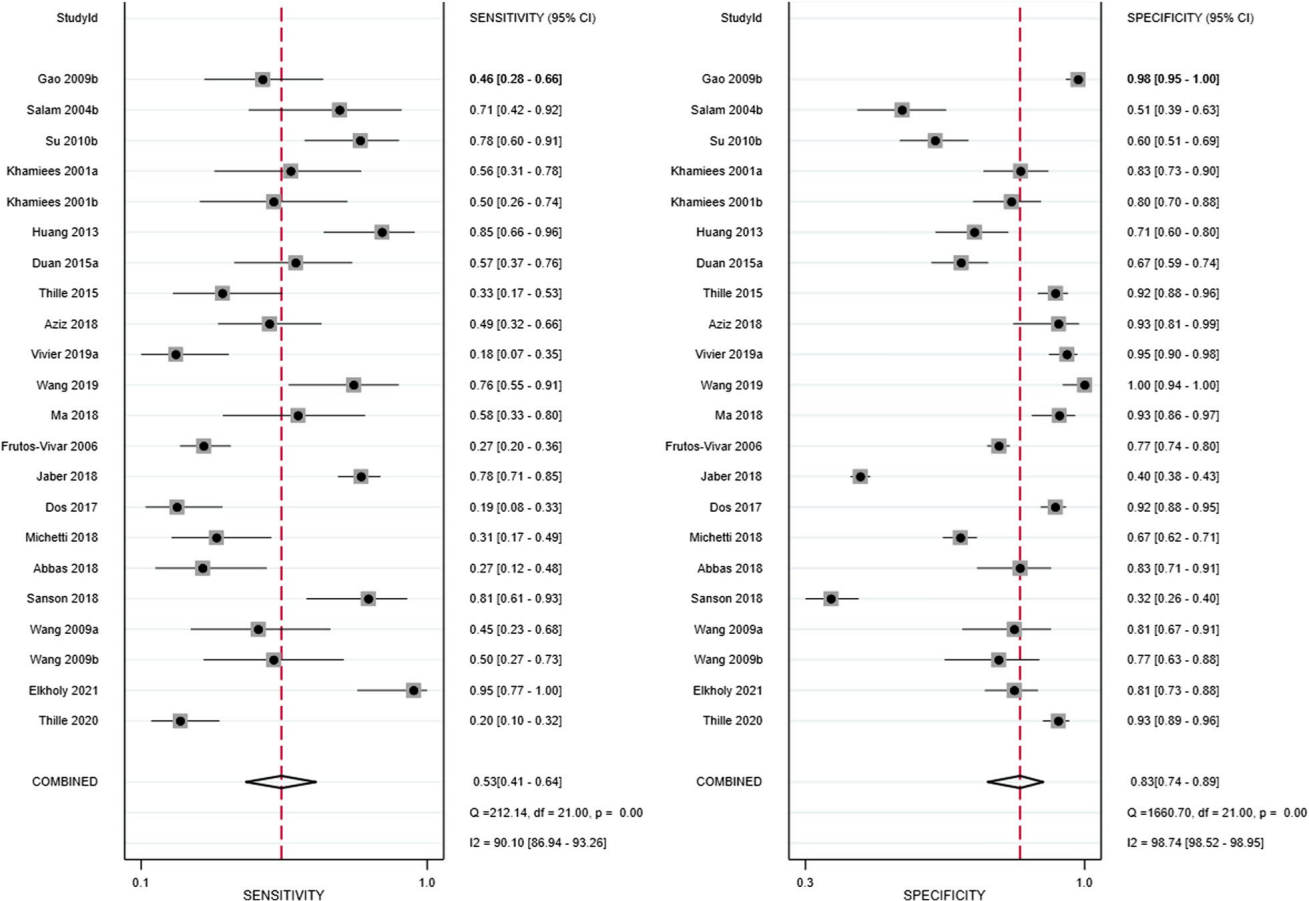
Forest plot of sensitivity and specificity in the diagnosis of extubation failure tested by the semiquantitative cough strength score. CI = confidence interval

**Fig. 6:**
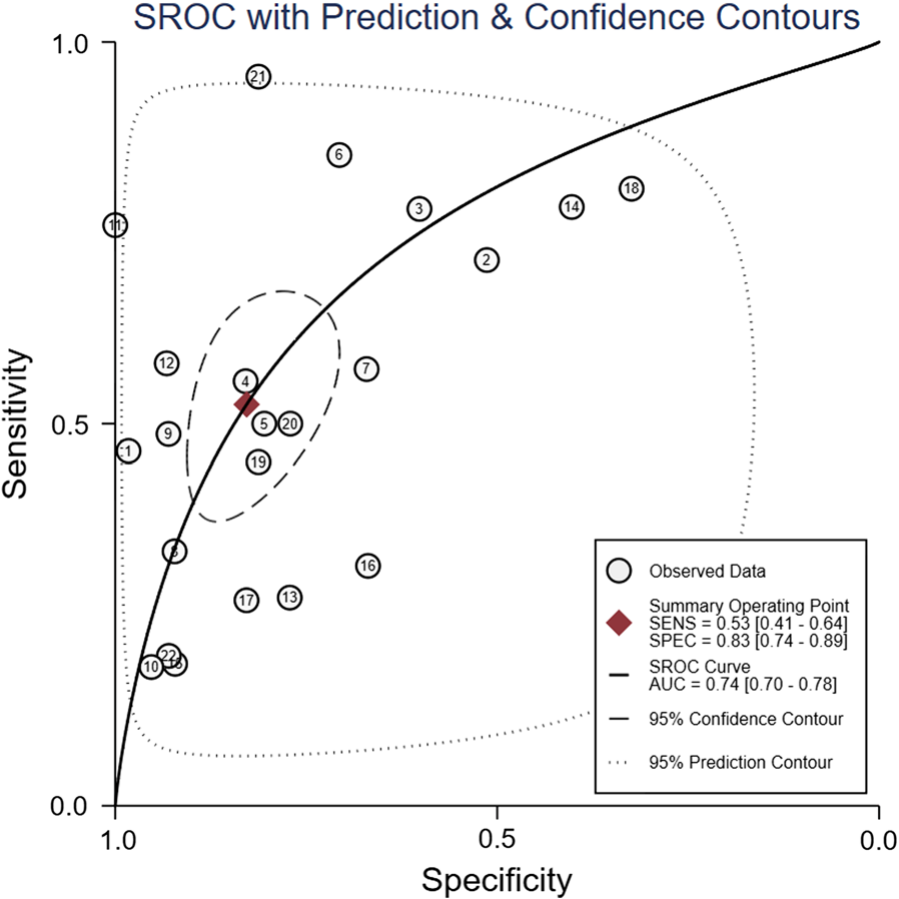
Summary receiver operator characteristic (SROC) curve in the prediction of extubation failure tested by the semiquantitative cough strength score. SENS = sensitivity, SPEC = specificity, AUC = area under the receiver operating characteristic curve. Numbers 1 to 22 represent the study arms (Gao 2009b, Salam 2004b, Su 2010b, Khamiees 2001a, Khamiees 2001b, Huang 2013, Duan 2015a, Thille 2015, Aziz 2018, Vivier 2019a, Wang 2019, Ma 2018, Frutos-Vivar 2006, Jaber 2018, Dos 2017, Michetti 2018, Abbas 2018, Sanson 2018, Wang 2009a, Wang 2009b, Elkholy 2021, and Thille 2020)

The pooled sensitivity and specificity were 0.53 (95% CI: 0.41–0.64) and 0.83 (0.74–0.89), respectively (Fig. 5). Meta-regression analyses indicated that sensitivity and specificity did not vary by publication year, country, study design, method used to assess the SCSS, number of cases in the study arm, time to extubation failure after the removal of the endotracheal tube, or definition of extubation failure (Additional file 9: Figure 9). The pooled positive LR and negative LR were 2.50 (95% CI: 1.93–3.25) and 0.65 (0.56–0.76), respectively (Additional file 10: Figure 10). The pooled DOR was 4.61 (95% CI: 3.03–7.01; Additional file 11: Figure 11). The AUC was 0.74 (95% CI: 0.70–0.78) when the SCSS was used to predict extubation failure (Fig. 6). The results for cough strength assessed by the SCSS graded from 0 to 4/5, the white card test (WCT), and other semiquantitative scales are summarised in Table 2.

## Discussion

To the best of our knowledge, this is the first systematic review and meta-analysis to explore the prediction of extubation failure diagnosed by cough strength. Cough peak flow includes voluntary and involuntary peak flow and can be measured with an external flowmeter or a ventilator. The SCSS can be measured with a scale from 0 to 4/5, the WCT, or other semiquantitative scales. Both cough peak flow and the SCSS show moderate diagnostic power for predicting extubation failure. However, cough peak flow is superior to the SCSS for predicting extubation failure.

Cough strength is strongly associated with maximal inspiratory and expiratory pressure [46], which in turn can reflect respiratory muscle function. Better respiratory muscle function is associated with lower extubation failure [47]. Therefore, weaker cough strength is associated with higher extubation failure. The current study with its large sample size demonstrates that both cough peak flow and the SCSS have moderate diagnostic power for predicting extubation failure. Therefore, cough strength can be commonly used to predict extubation failure in clinical practice.

Cough peak flow includes voluntary and involuntary peak flow. Voluntary peak flow can be measured when the investigator coaches the patient to cough. Involuntary peak flow can be stimulated with an injection of 2 mL normal saline or with a suction catheter. Two studies measured both voluntary and involuntary peak flow. One showed that voluntary peak flow was better than involuntary peak flow at predicting extubation failure [11]. However, the other showed no difference between the two methods in predicting extubation failure [30]. The current meta-analysis, which enrolled 17 study arms that measured voluntary peak flow and 6 that measured involuntary peak flow, found that involuntary peak flow had much higher predictive power than voluntary peak flow. Voluntary peak flow can only be measured in cooperative patients, as it requires the patient to cough on command. However, involuntary peak flow can be measured in all patients, even unconscious patients, as it does not require the patient’s cooperation. Thus, involuntary peak flow may be more suitable for predicting extubation failure in patients who are ready for extubation.

Cough peak flow can be measured with an external flowmeter or a ventilator. Only one study with 126 cases measured cough peak flow using both methods [26]. And both methods showed similar predictive accuracy. However, given the small sample size in that study, its power is inadequate. Our meta-analysis, which enrolled 18 study arms that measured cough peak flow with an external flowmeter and 5 that measured it with a ventilator, found that the AUC was higher when cough peak flow was measured with an external flowmeter than a ventilator. This indicates that predictive accuracy is greater when cough peak flow is measured with an external flowmeter. However, measuring cough peak flow with an external flowmeter requires a dedicated device. This may limit the use of this method. As the AUC was 0.77 when cough peak flow was measured with a ventilator, indicating moderate accuracy for predicting extubation failure, it can be used to predict extubation failure if an external flowmeter is unavailable. However, cut-off values differ among studies. This may be related to the different devices used in the studies. Therefore, the generalisation of the measure of cough peak flow is limited by the variability in cut-off values by study, even when the method is the same.

The SCSS, which ranges from 0 to 4/5, was the most common semiquantitative method of measuring cough strength in this meta-analysis. A score of 0 indicates the weakest cough, and a score of 4/5 indicates the strongest cough [18, 21]. The WCT was another semiquantitative method used to measure cough strength [13]. However, no studies compared the two methods on their predictive accuracy for extubation failure. This study found that the WCT is more accurate than an SCSS score of 0–4/5 for predicting extubation failure. The SCSS graded 0–4/5 is subjectively rated by the investigators. However, the WCT, which is scored based on the moisture on a card when the investigator coaches the patient to cough, is less likely to be influenced by the investigator’s experience. Thus, the WCT can be given priority over the SCSS for predicting extubation failure.

Sensitivity was lower but specificity was higher when the SCSS (vs. cough peak flow) was used to assess cough strength. This might suggest that weak cough identified using the SCSS is actually very weak with a very low peak flow (if performed) and consequently associated with more false negatives but fewer false positives. When patients are identified as having weak cough using the SCSS, their risk of extubation failure is very high. In contrast, patients identified as having weak cough using peak flow may have a stronger cough than those identified as having weak cough using the SCSS and consequently fewer false negatives and more false positives. It may be that the SCSS is unable to detect weak cough in patients with moderately decreased peak flow (around 60 L/min).

This study has several limitations. First, the time between the removal of the endotracheal tube and extubation failure was the main high risk of quality evaluation on included studies. However, we analysed studies that defined extubation failure within and beyond 72 h. The meta-regression showed that this factor did not influence sensitivity and specificity. Second, publication bias was observed among studies that measured the SCSS. We performed a sensitivity analysis and found that the pooled DOR ranged from 4.08 to 5.02 and the pooled AUC ranged from 0.71 to 0.75. This indicates that the results were stable despite the presence of publication bias. Third, judging weak cough is difficult, as the definition of weak cough varies by study. A consensus on the definition of weak cough based on cough peak flow or the SCSS would be helpful for improving operability. Fourth, different types of SBTs were performed in the enrolled studies. The rate of successful SBTs was higher when they were performed under pressure support ventilation than under T-piece or continuous positive airway pressure [48]. However, extubation failure did not vary by type of SBT [49, 50]. Therefore, type of SBT is unlikely to influence results for the association between cough strength and extubation failure.

## Conclusions

Weak cough is associated with increased extubation failure. It can be assessed by cough peak flow and the SCSS. The predictive power of cough peak flow may be better than that of the SCSS for diagnosing extubation failure.

## Supplementary Information

**Additional file 1**: Figure 1. Pooled extubation failure in patients with weak and strong cough tested by cough peak flow (CPF). CI = confidence interval.

**Additional file 2**: Figure 2. Pooled extubation failure in patients with weak and strong cough tested by the semiquantitative cough strength score (SCSS). CI = confidence interval.

**Additional file 3**: Figure 3. Deeks’ funnel plot of publication bias among studies that assessed cough peak flow. ESS = effective sample size. Numbers 1 to 23 represent the study arms (Beuret 2009, Duan 2014a, Duan 2014b, Gao 2009a, Salam 2004a, Smailes 2013, Smina 2003, Su 2010a, Gobert 2017, Liu 2014, Duan 2015b, Bai 2017a, Bai 2017b, Xiao 2018, Duan 2017, Kutchak 2015, Almeida 2020a, Almeida 2020b, Almeida 2020c, Vivier 2019b, Norisue 2020, Lu 2010, and Liang 2019).

**Additional file 4**: Figure 4. Deeks’ funnel plot of publication bias among studies that assessed the semiquantitative cough strength score. ESS = effective sample size. Numbers 1 to 22 represent the study arms (Gao 2009b, Salam 2004b, Su 2010b, Khamiees 2001a, Khamiees 2001b, Huang 2013, Duan 2015a, Thille 2015, Aziz 2018, Vivier 2019a, Wang 2019, Ma 2018, Frutos-Vivar 2006, Jaber 2018, Dos 2017, Michetti 2018, Abbas 2018, Sanson 2018, Wang 2009a, Wang 2009b, Elkholy 2021, and Thille 2020).

**Additional file 5**: Figure 5. Sensitivity analysis of the diagnostic odds ratio (DOR) and area under the receiver operating characteristic curve (AUC) among studies that assessed the semiquantitative cough strength score when one study arm was omitted.

**Additional file 6**: Figure 6. Meta-regression analysis of studies that assessed cough peak flow (CPF). CI = confidence interval. Meta-regression was performed by publication year, country (China, France, USA, or other), voluntary or involuntary CPF, assessment of CPF with an external flowmeter or a ventilator, different cut-off values, number of cases in the study arm, time to extubation failure (EF) after the removal of the endotracheal tube (≤72 h or >72 h), and definition of EF (reintubation, death, or noninvasive ventilation).

**Additional file 7**: Figure 7. Forest plot of the positive likelihood ratio (LR) and negative LR in the diagnosis of extubation failure tested by cough peak flow. CI = confidence interval.

**Additional file 8**: Figure 8. Forest plot of the diagnostic odds ratio (OR) in the prediction of extubation failure tested by cough peak flow. CI = confidence interval.

**Additional file 9**: Figure 9. Meta-regression analysis of studies that assessed the semiquantitative cough strength score (SCSS). CI = confidence interval. Meta-regression was performed by publication year, country (China, France, USA, or other), study design (prospective or retrospective), method of measuring the SCSS (white card test or not), number of cases in the study arm, time to extubation failure (EF) after the removal of the endotracheal tube (≤72 h or >72 h), and definition of EF (reintubation, death, or noninvasive ventilation).

**Additional file 10**: Figure 10. Forest plot of the positive likelihood ratio (LR) and negative LR in the diagnosis of extubation failure tested by the semiquantitative cough strength score. CI = confidence interval.

**Additional file 11**: Figure 11. Forest plot of the diagnostic odds ratio (OR) in the prediction of extubation failure tested by the semiquantitative cough strength score. CI = confidence interval.

**Additional file 12**. Details on the different subgroups.

## Data Availability

All data generated and/or analysed during the current study are included within the published article and its additional files.

## Abbreviations

MV: Mechanical ventilation
SBT: Spontaneous breathing trial
CPF: Cough peak flow
SCSS: Semiquantitative cough strength score
WCT: White card test
TP: True positive
FP: False positive
FN: False negative
TN: True negative
LR: Likelihood ratio
DOR: Diagnostic odds ratio
AUC: Area under the receiver operating characteristic curves
CI: Confidence interval
SENS: Sensitivity
SPEC: Specificity
SROC: Summary receiver operator characteristic
ESS: Effective sample size
