# Supplementary material for: Predictive power of extubation failure diagnosed by cough strength: a systematic review and meta-analysis
Source: Crit Care. 2021 Oct 12;25:357. doi: 10.1186/s13054-021-03781-5 (PMC8513306; doi:10.1186/s13054-021-03781-5)
Supplement: Supplementary file 1 — Additional file 1: Figure 1. Pooled extubation failure in patients with weak and strong cough tested by cough peak flow (CPF). CI = confidence interval. [file 13054_2021_3781_MOESM1_ESM.pdf]

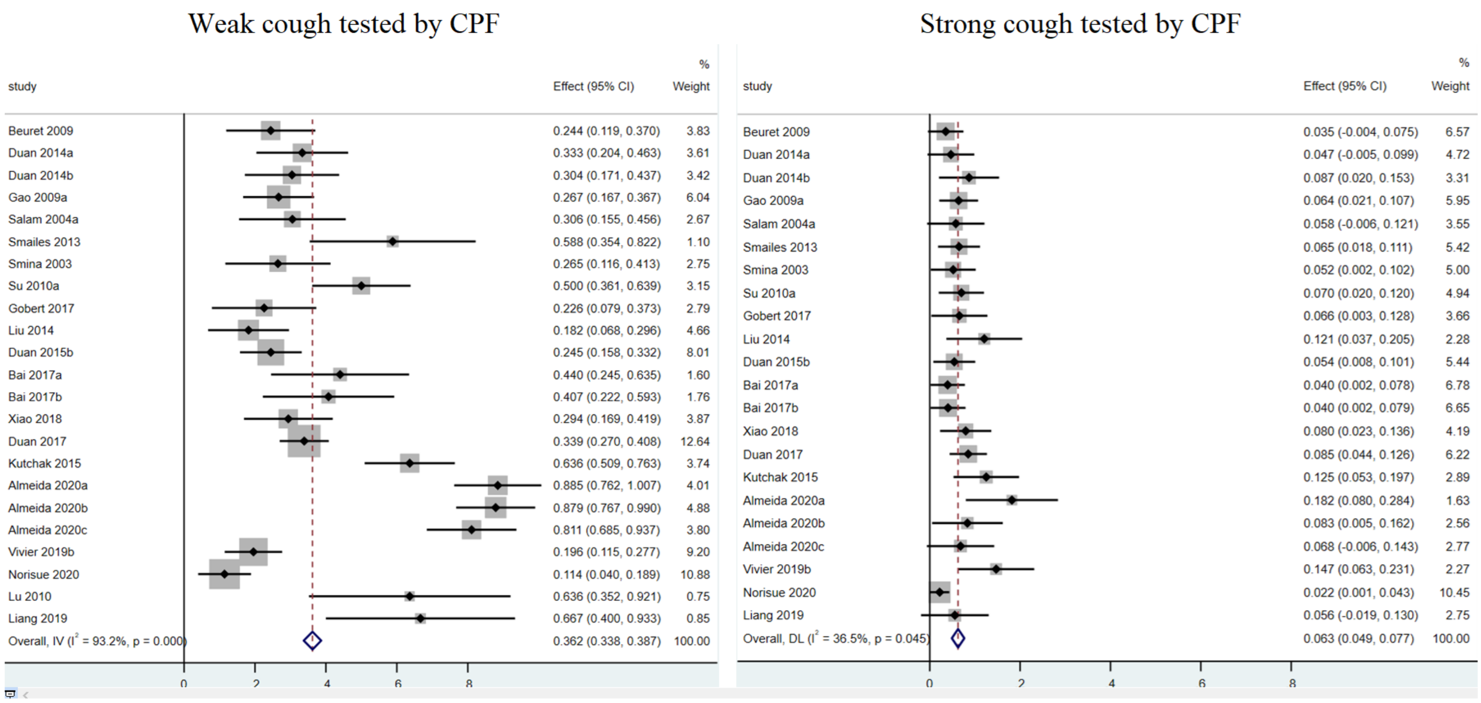

Supplementary Figure 1. Pooled extubation failure in patients with weak and strong cough tested by cough peak flow (CPF). CI = confidence interval
