# Supplementary material for: Predictive power of extubation failure diagnosed by cough strength: a systematic review and meta-analysis
Source: Crit Care. 2021 Oct 12;25:357. doi: 10.1186/s13054-021-03781-5 (PMC8513306; doi:10.1186/s13054-021-03781-5)
Supplement: Supplementary file 3 — Additional file 3: Figure 3. Deeks’ funnel plot of publication bias among studies that assessed cough peak flow. ESS = effective sample size. Numbers 1 to 23 represent the study arms (Beuret 2009, Duan 2014a, Duan 2014b, Gao 2009a, Salam 2004a, Smailes 2013, Smina 2003, Su 2010a, Gobert 2017, Liu 2014, Duan 2015b, Bai 2017a, Bai 2017b, Xiao 2018, Duan 2017, Kutchak 2015, Almeida 2020a, Almeida 2020b, Almeida 2020c, Vivier 2019b, Norisue 2020, Lu 2010, and Liang 2019). [file 13054_2021_3781_MOESM3_ESM.pdf]

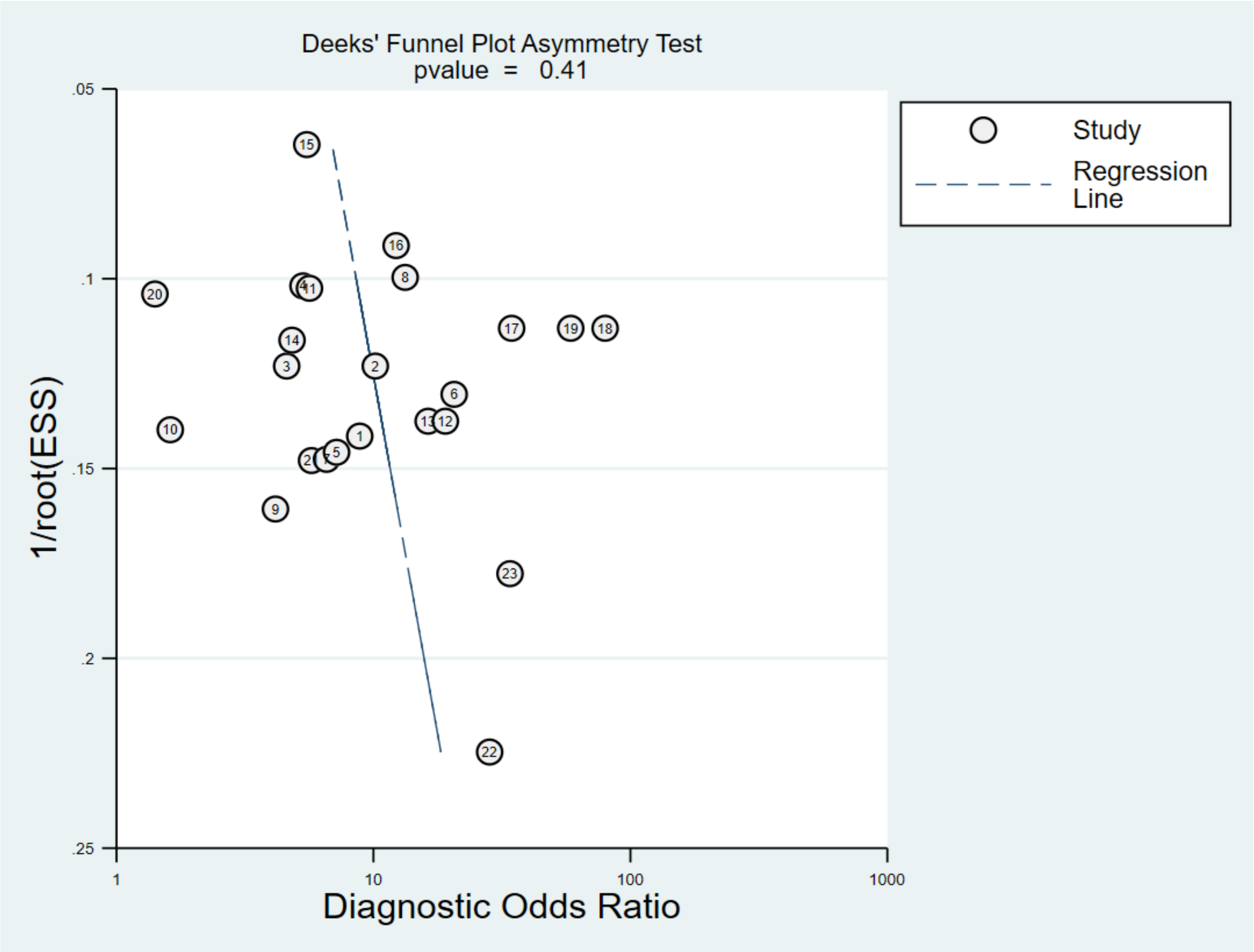

Supplementary Figure 3. Deeks' funnel plot of publication bias among studies that assessed cough peak flow. ESS = effective sample size. Numbers 1 to 23 represent the study arms (Beuret 2009, Duan 2014a, Duan 2014b, Gao 2009a, Salam 2004a, Smailes 2013, Smina 2003, Su 2010a, Gobert 2017, Liu 2014, Duan 2015b, Bai 2017a, Bai 2017b, Xiao 2018, Duan 2017, Kutchak 2015, Almeida 2020a, Almeida 2020b, Almeida 2020c, Vivier 2019b, Norisue 2020, Lu 2010, and Liang 2019).
