# Supplementary material for: Predictive power of extubation failure diagnosed by cough strength: a systematic review and meta-analysis
Source: Crit Care. 2021 Oct 12;25:357. doi: 10.1186/s13054-021-03781-5 (PMC8513306; doi:10.1186/s13054-021-03781-5)
Supplement: Supplementary file 4 — Additional file 4: Figure 4. Deeks’ funnel plot of publication bias among studies that assessed the semiquantitative cough strength score. ESS = effective sample size. Numbers 1 to 22 represent the study arms (Gao 2009b, Salam 2004b, Su 2010b, Khamiees 2001a, Khamiees 2001b, Huang 2013, Duan 2015a, Thille 2015, Aziz 2018, Vivier 2019a, Wang 2019, Ma 2018, Frutos-Vivar 2006, Jaber 2018, Dos 2017, Michetti 2018, Abbas 2018, Sanson 2018, Wang 2009a, Wang 2009b, Elkholy 2021, and Thille 2020). [file 13054_2021_3781_MOESM4_ESM.pdf]

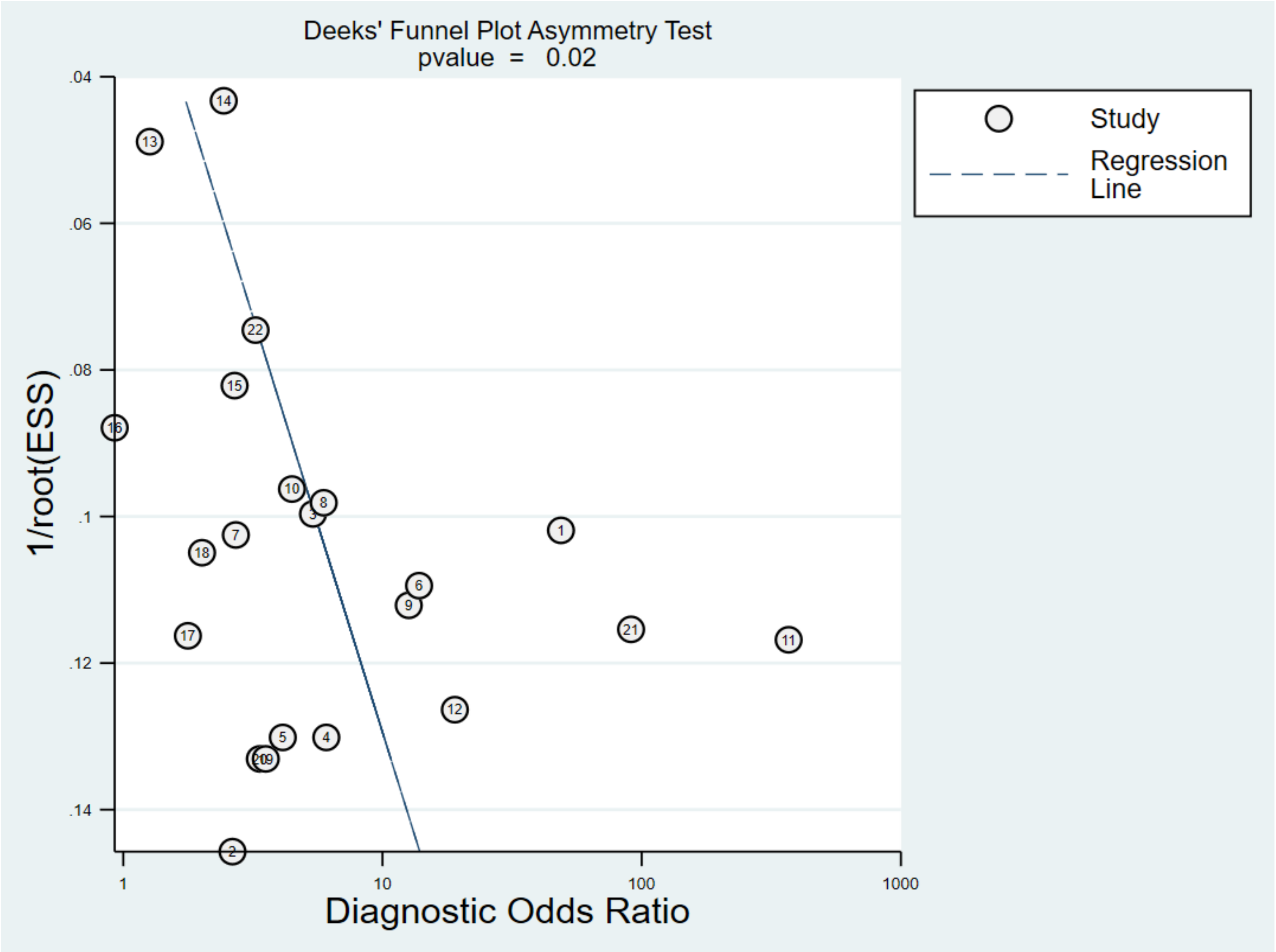


**Supplementary Figure 4.** Deeks' funnel plot of publication bias among studies that assessed the semiquantitative cough strength score. ESS = effective sample size. Numbers 1 to 22 represent the study arms (Gao 2009b, Salam 2004b, Su 2010b, Khamiees 2001a, Khamiees 2001b, Huang 2013, Duan 2015a, Thille 2015, Aziz 2018, Vivier 2019a, Wang 2019, Ma 2018, Frutos-Vivar 2006, Jaber 2018, Dos 2017, Michetti 2018, Abbas 2018, Sanson 2018, Wang 2009a, Wang 2009b, Elkholy 2021, and Thille 2020).
