# Supplementary material for: Predictive power of extubation failure diagnosed by cough strength: a systematic review and meta-analysis
Source: Crit Care. 2021 Oct 12;25:357. doi: 10.1186/s13054-021-03781-5 (PMC8513306; doi:10.1186/s13054-021-03781-5)
Supplement: Supplementary file 5 — Additional file 5: Figure 5. Sensitivity analysis of the diagnostic odds ratio (DOR) and area under the receiver operating characteristic curve (AUC) among studies that assessed the semiquantitative cough strength score when one study arm was omitted. [file 13054_2021_3781_MOESM5_ESM.pdf]

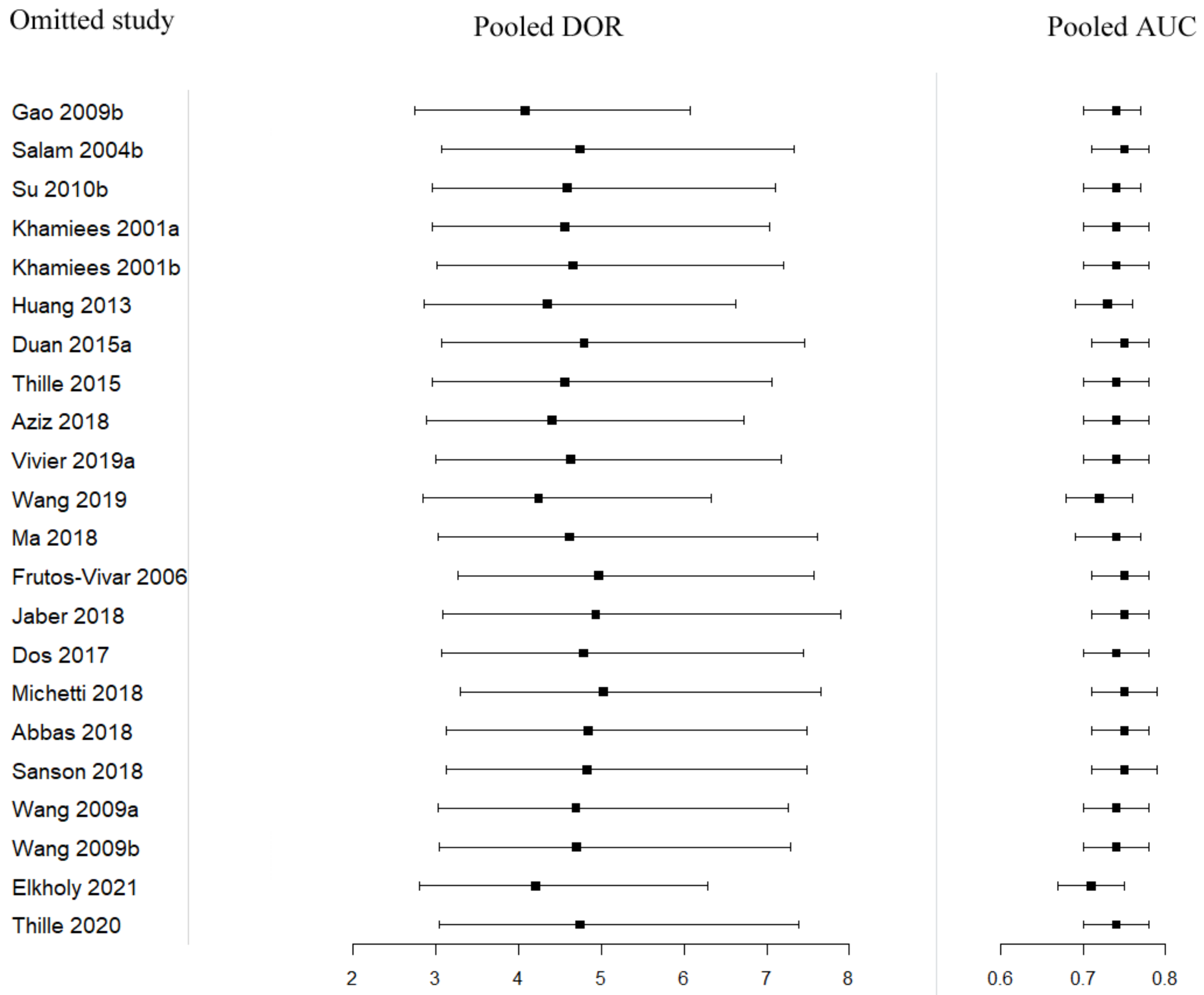

Supplementary Figure 5. Sensitivity analysis of the diagnostic odds ratio (DOR) and area under the receiver operating characteristic curve (AUC) among studies that assessed the semiquantitative cough strength score when one study arm was omitted.
