# Supplementary material for: Predictive power of extubation failure diagnosed by cough strength: a systematic review and meta-analysis
Source: Crit Care. 2021 Oct 12;25:357. doi: 10.1186/s13054-021-03781-5 (PMC8513306; doi:10.1186/s13054-021-03781-5)
Supplement: Supplementary file 6 — Additional file 6: Figure 6. Meta-regression analysis of studies that assessed cough peak flow (CPF). CI = confidence interval. Meta-regression was performed by publication year, country (China, France, USA, or other), voluntary or involuntary CPF, assessment of CPF with an external flowmeter or a ventilator, different cut-off values, number of cases in the study arm, time to extubation failure (EF) after the removal of the endotracheal tube (≤72 h or >72 h), and definition of EF (reintubation, death, or noninvasive ventilation). [file 13054_2021_3781_MOESM6_ESM.pdf]

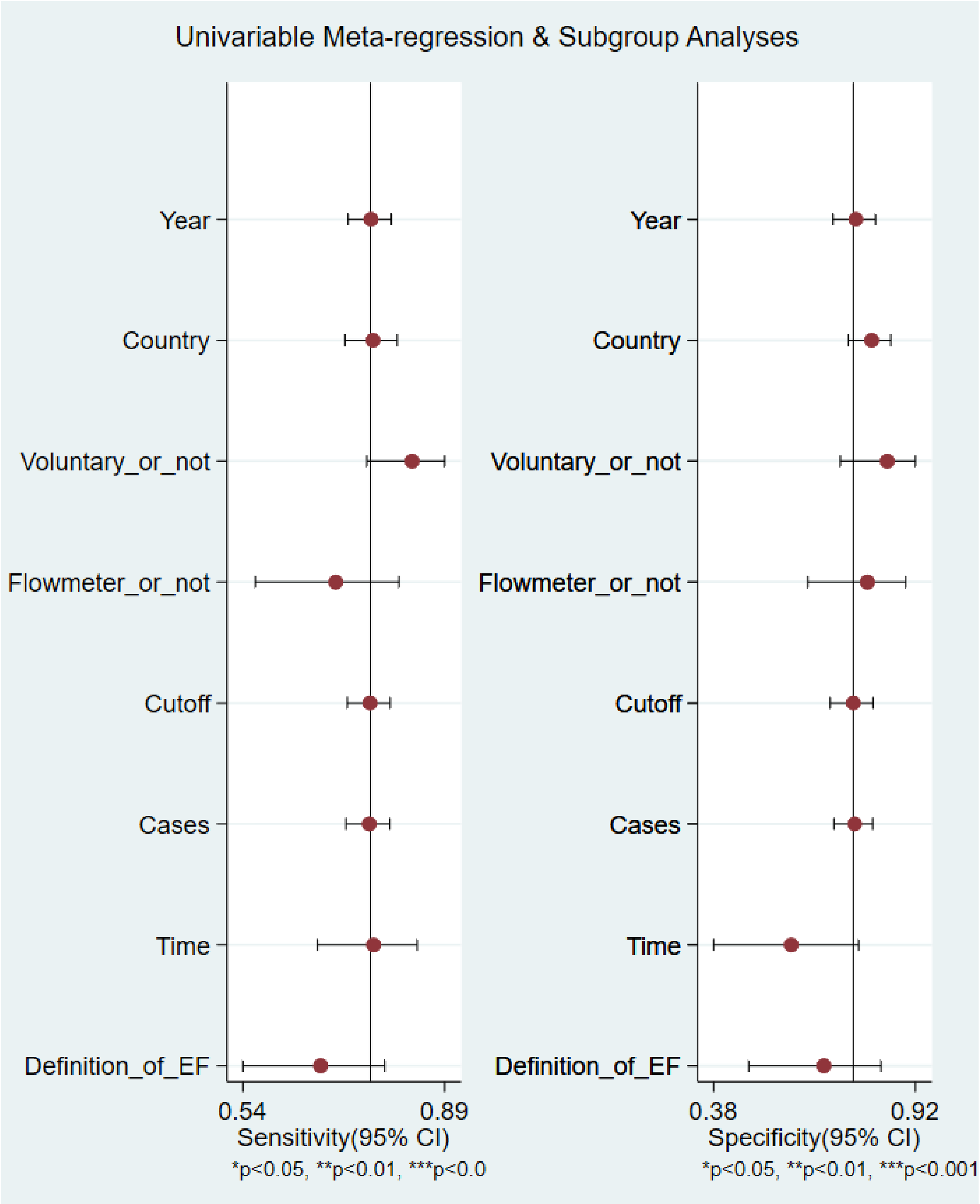

Supplementary Figure 6. Meta-regression analysis of studies that assessed cough peak flow (CPF). CI = confidence interval. Meta-regression was performed by publication year, country (China, France, United States, or other), voluntary or involuntary CPF, assessment of CPF with an external flowmeter or a ventilator, different cutoff values, number of cases in the study arm, time to extubation failure (EF) after the removal of the endotracheal tube (≤72 h or >72 h), and definition of EF (reintubation, death, or noninvasive ventilation).
