# Supplementary material for: Predictive power of extubation failure diagnosed by cough strength: a systematic review and meta-analysis
Source: Crit Care. 2021 Oct 12;25:357. doi: 10.1186/s13054-021-03781-5 (PMC8513306; doi:10.1186/s13054-021-03781-5)
Supplement: Supplementary file 9 — Additional file 9: Figure 9. Meta-regression analysis of studies that assessed the semiquantitative cough strength score (SCSS). CI = confidence interval. Meta-regression was performed by publication year, country (China, France, USA, or other), study design (prospective or retrospective), method of measuring the SCSS (white card test or not), number of cases in the study arm, time to extubation failure (EF) after the removal of the endotracheal tube (≤72 h or >72 h), and definition of EF (reintubation, death, or noninvasive ventilation). [file 13054_2021_3781_MOESM9_ESM.pdf]

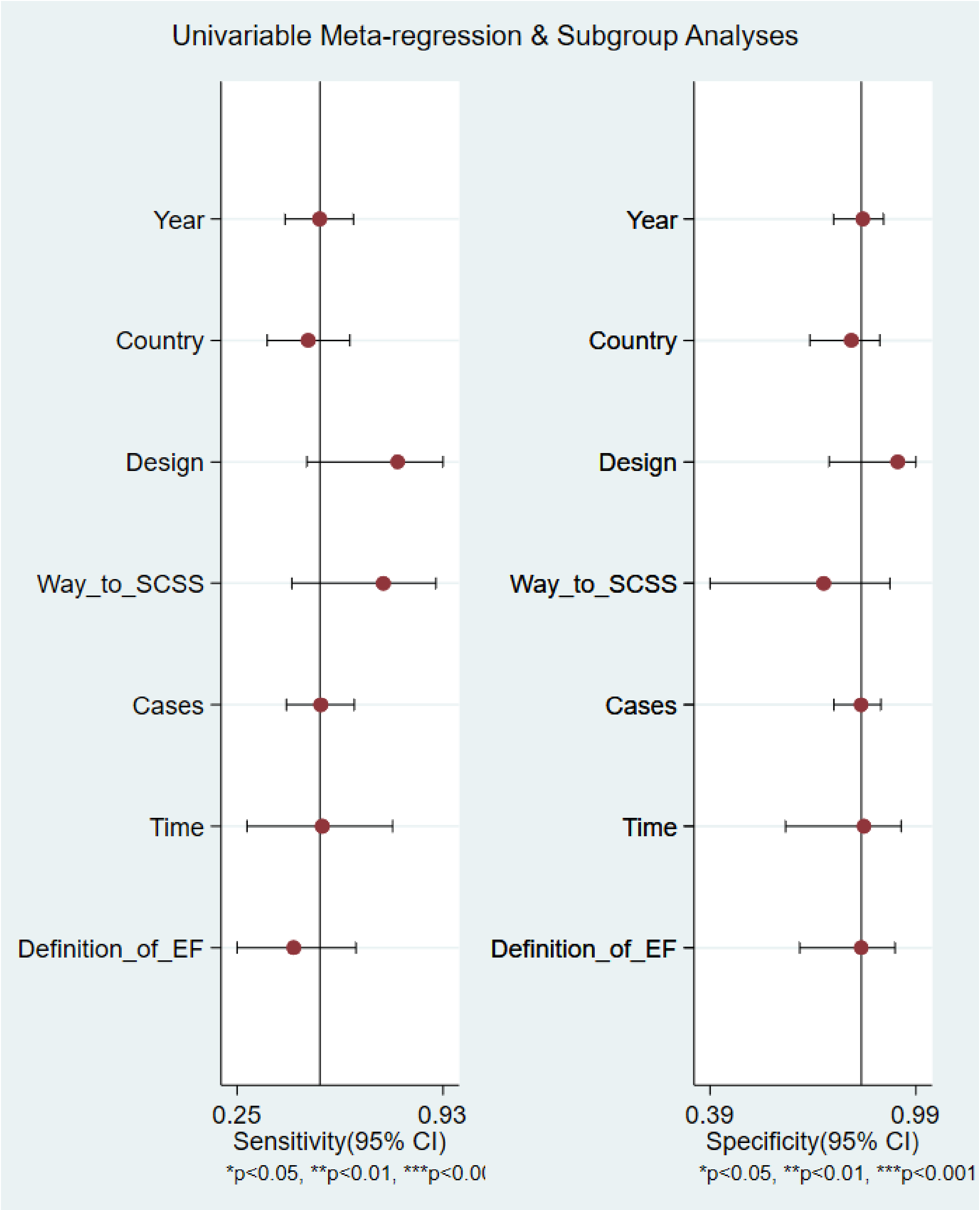

Supplementary Figure 9. Meta-regression analysis of studies that assessed the semiquantitative cough strength score (SCSS). CI = confidence interval. Meta-regression was performed by publication year, country (China, France, United States, or other), study design (prospective or retrospective), method of measuring the SCSS (white card test or not), number of cases in the study arm, time to extubation failure (EF) after the removal of the endotracheal tube (≤72 h or >72 h), and definition of EF (reintubation, death, or noninvasive ventilation).
