# Supplementary material for: Predictive power of extubation failure diagnosed by cough strength: a systematic review and meta-analysis
Source: Crit Care. 2021 Oct 12;25:357. doi: 10.1186/s13054-021-03781-5 (PMC8513306; doi:10.1186/s13054-021-03781-5)
Supplement: Supplementary file 10 — Additional file 10: Figure 10. Forest plot of the positive likelihood ratio (LR) and negative LR in the diagnosis of extubation failure tested by the semiquantitative cough strength score. CI = confidence interval. [file 13054_2021_3781_MOESM10_ESM.pdf]

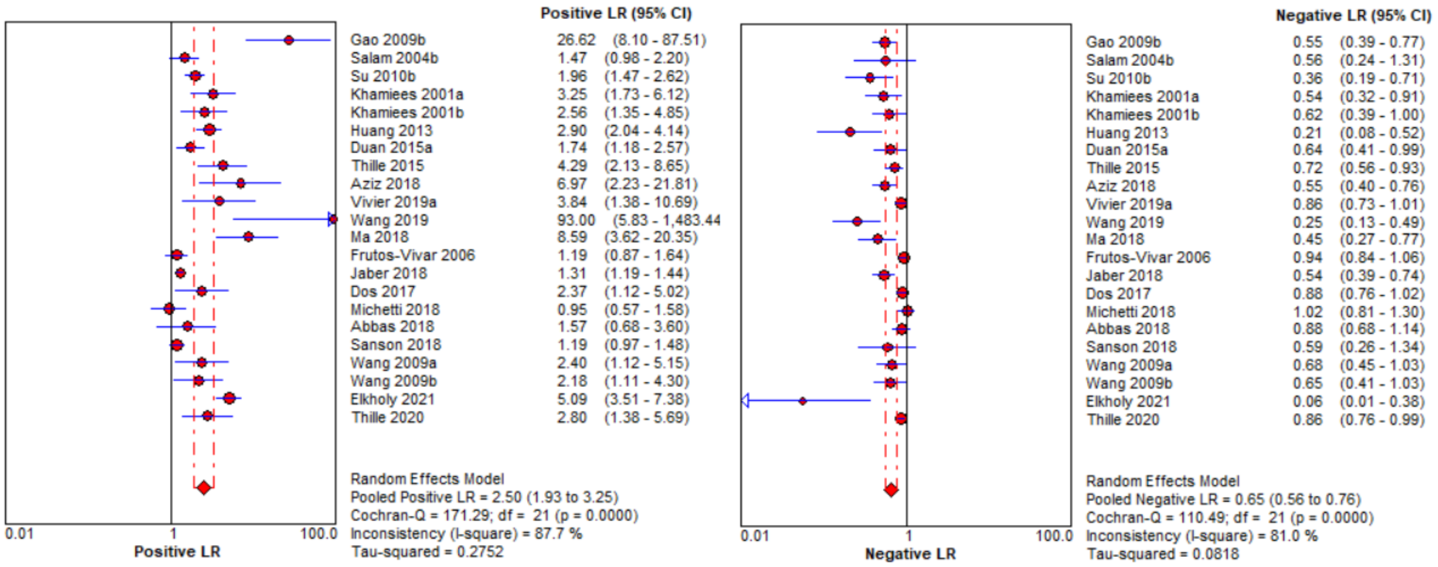

Supplementary Figure 10. Forest plot of the positive likelihood ratio (LR) and negative LR in the diagnosis of extubation failure tested by the semiquantitative cough strength score. CI = confidence interval
