# Supplementary material for: Predictive power of extubation failure diagnosed by cough strength: a systematic review and meta-analysis
Source: Crit Care. 2021 Oct 12;25:357. doi: 10.1186/s13054-021-03781-5 (PMC8513306; doi:10.1186/s13054-021-03781-5)
Supplement: Supplementary file 12 — Additional file 12. Details on the different subgroups. [file 13054_2021_3781_MOESM12_ESM.doc]

**Supplementary Text 1. Details on the different subgroups**

Four subgroups among the studies measured cough peak flow (CPF). Details are reported in Table 2. Seventeen study arms involving 2408 tests measured voluntary CPF, which was tested when the investigator coached the patient to cough as hard as possible. Six study arms involving 610 tests measured involuntary CPF, which was tested when a cough was stimulated with 2 mL normal saline or suction catheter. Eighteen study arms involving 2300 tests measured CPF with an external flowmeter. Five study arms involving 718 tests measured CPF with a ventilator.

Three subgroups among the studies measured the semiquantitative cough strength score (SCSS). Details are reported in Table 2. Eight studies involving 1342 tests measured the SCSS ranging from 0 to 4/5 (weakest to strongest). Weak cough was defined as SCSS = 0 to 1/2. Four studies involving 406 tests measured the SCSS with the white card test (WCT). The WCT was performed before extubation. The investigator coached the patient to cough through an open-ended endotracheal tube while a white file card was placed 1–2 cm from its end. A lack of moisture on the card after two or three coughs was considered a negative WCT result and was defined as weak cough. The other 10 study arms involving 3918 tests made up the third subgroup. The definition of weak cough varied in this subgroup.
